# Socio-Economic Context and Community Resilience among the People Involved in Fish Drying Practices in the South-East Coast of Bangladesh

**DOI:** 10.3390/ijerph18126242

**Published:** 2021-06-09

**Authors:** Sabrina Jannat Mitu, Petra Schneider, Md. Shahidul Islam, Masud Alam, Mohammad Mojibul Hoque Mozumder, Mohammad Mosarof Hossain, Md. Mostafa Shamsuzzaman

**Affiliations:** 1 Department of Coastal and Marine Fisheries, Sylhet Agricultural University, Sylhet 3100, Bangladesh; mitusabrina42@gmail.com (S.J.M.); islamms2011@yahoo.com (M.S.I.); mosarofsau@gmail.com (M.M.H.); 2 Department for Water, Environment, Civil Engineering and Safety, University of Applied Sciences, Magdeburg-Stendal, Breitscheidstraße 2, D-39114 Magdeburg, Germany; petra.schneider@h2.de; 3 Department of Agricultural Statistics, Sylhet Agricultural University, Sylhet 3100, Bangladesh; malam.stat@sau.ac.bd; 4 Fisheries and Environmental Management Group, Helsinki Institute of Sustainability Science (HELSUS), Faculty of Biological and Environmental Sciences, University of Helsinki, 00014 Helsinki, Finland; mohammad.mozumder@helsinki.fi

**Keywords:** coastal fisheries, dry fish, livelihood, vulnerability, AHP

## Abstract

The south-east coast, specifically the Cox’s Bazar region, of Bangladesh has achieved a tremendous impetus for producing a large volume of dried fish by involving thousands of marginalized coastal people. This study aimed to assess the socio-economic profile, livelihood strategies, and resilience of the communities engaged in fish drying on the south-east coast using a mixed-methods approach and an Analytic Hierarchy Process (AHP). The study’s findings revealed that communities involved in drying were socio-economically undeveloped due to their lower literacy, unstable incomes, and labor-intensive occupations. Apart from notable child labor employed in fish drying in Nazirertek, female workers had relatively higher participation than males. Nevertheless, the female workers had less control over their daily wages and reported working at USD 3.54–5.89 per day, which was relatively lower than male workers who received USD 4.15–8.31 per day. Through fish drying activities, very few workers, producers, and traders were found to be self-reliant. In contrast, the livelihoods of the workers were not as secure as the processors and traders. In addition to suffering from various shocks and constraints, dried fish processors and workers, dried fish traders, off-season income, an abundance of fish species, fish drying facilities, trader’s association, and social interrelationship played a significant role in maintaining community resilience. The study recommends appropriate interventions to alternative income diversification options, strong collaboration between communities, local authorities, and government for sustainable livelihoods and better community resilience.

## 1. Introduction

Dried fish accounts for the 4th most significant share of fish consumed in Bangladesh [1] and is much relished by the country’s people for its flavor, texture, and taste [2]. It is an accessible and low-cost food source and can contribute a large percentage of protein and significant micronutrients to the diet of poor people [3]. Fish drying is the most extensive fish processing activity in Bangladesh’s coastal region that contributes significantly to livelihoods and nutrition, especially for poor and marginalized communities in coastal and inland areas [4]. These activities are of great importance to Bangladesh, as more than 17 million people, including 1.4 million women, depend on fish farming, processing, and handling [5]. After harvesting, more than one-third of the landings are used for drying all year round [6,7]; therefore, these drying practices have provided solvency to thousands of coastal populations.

The processing and trade of dried fish are becoming a promising and profitable industry, offering the processors, traders, and other stakeholders opportunities to make much money in the fisheries sector. As a result, dried fish has demand both on the national and international markets. In contrast, the export of dried fishes has increased from 517 metric tons (value 94 million USD) in the fiscal year 2001–2002 to 3144 metric tons (value 5.01 million USD) in 2018–2019 [8]. However, in the context of global food security and livelihoods of small-scale fishers in developing countries, it is dispiriting that the importance of dried fish and the people, directly and indirectly, involved in drying are poorly understood and rarely recognized [1]. Therefore, the study of the socio-economic condition and community’s resilience is essential. Moreover, it explains the actual situation of the population in a particular region and allows seeing how individuals or families fit into society through economic and social measures. Finally, such studies help to take appropriate initiatives for the proper management of communities.

Fish drying activities on the south-east coast vary considerably according to the weather conditions. Nevertheless, the negative impact of climate change is a severe concern at regional, national, and global levels, affecting most reclaims’ sustainability perspectives, including the aquatic environments, ecosystems, and the dependent societies [9,10,11,12]. The economies and livelihoods of communities dealing with dried fish’s processing and trade are affected by climate variability. In contrast, the communities are vulnerable to extreme weather conditions (tidal storms, heavy rains, cloudy weather) [13]. These situations raise the need to address sustainable livelihoods and community resilience among those engaged in fish drying. Hence, the purpose of this study is to assess the socio-economic conditions and the key indicators of strength for the communities involved in fish drying practices on the south-east coast of Bangladesh. Many studies are available on the socio-economic conditions of fishers in Bangladesh [14,15,16,17,18]. However, scanty research work has been done on the fish drying communities except for their socio-economic conditions, such as labor well-being’s in dried fish value chains [1], the efficiency of dry fish marketing [6], and quality analysis of dry fish [19]. The study’s findings will help the selected communities, different organizations, and government bodies to formulate policies for improving the socio-economic conditions of fish drying communities.

## 2. Materials and Methods

### 2.1. Profile of the Study Sites

**Figure 1:**
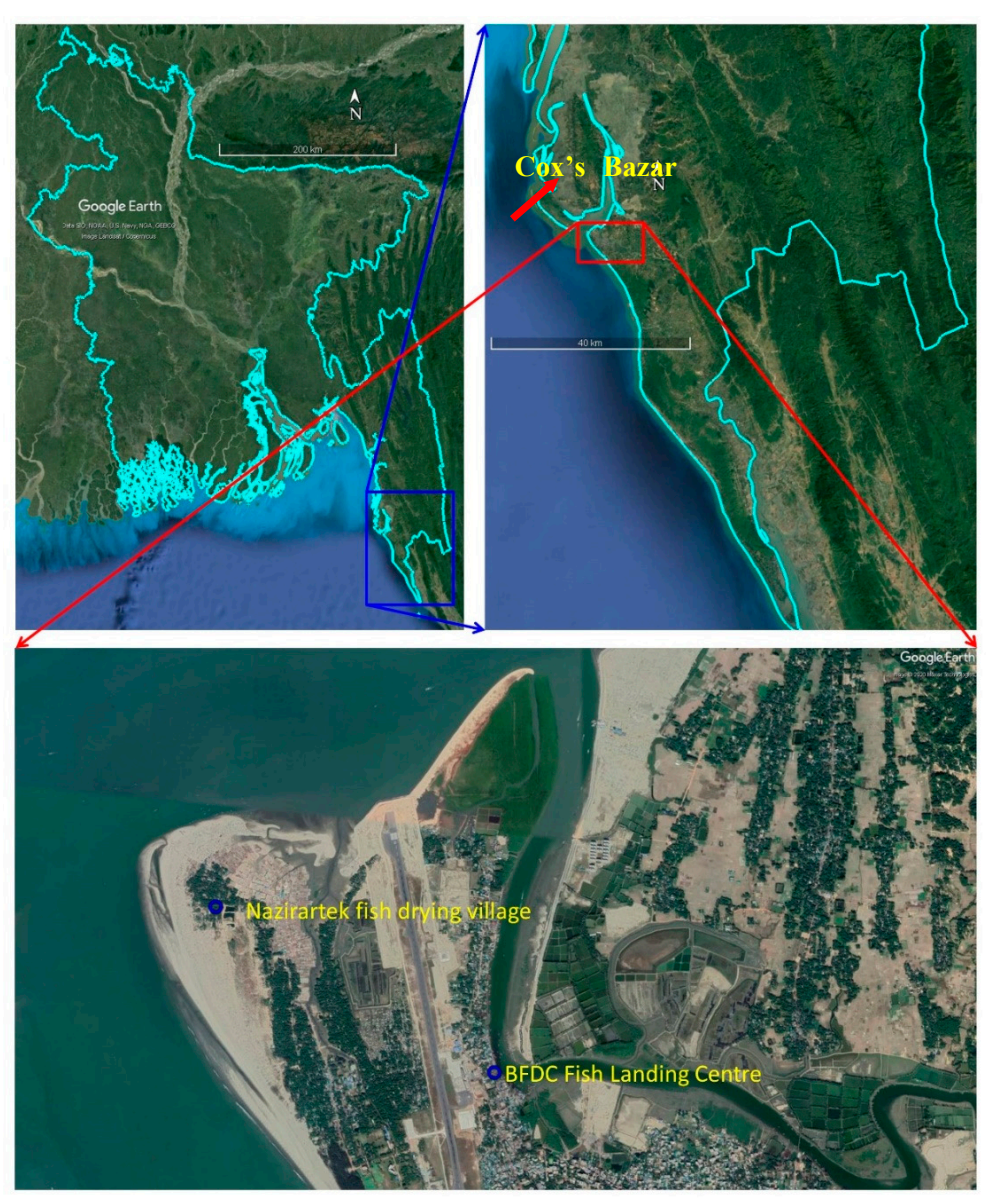
Location of the study area in Cox’s Bazar district of Bangladesh (Google Maps).

The study was carried out at two fish drying areas along the south-east coast: Nazirertek (under Ward no. 1 of Cox’s Bazar Sadar) and Chitapara (under Ward no. 2 of Cox’s Bazar Sadar) located in the Cox’s Bazar district of Bangladesh (Figure 1). The main criterion for selecting these two areas was the community’s reliance on fish drying.

Nazirertek (one of the most extensive fish drying yards in Bangladesh) has been built on approximately 200 acres of land at the Bhakkhali River’s mouth, Cox’s Bazar. Processing of dried fish begins in mid-August, and if the weather remains good, the process continues until mid-April/May of the following year (also called peak season). During the peak season, 20,000 workers (most of them women) work in different *Shutki mahals* (fish drying yards), and about 45.34−54.43 × 10^5^ metric tons of dried fish are produced in the *Shutki Palli* (fish drying village) at a market price of around USD 24 million [20]. Traditional solar energy methods are used for large-scale fish species drying, including Chhuri (*Trichiurus haumela)*, Laitta (*Harpadon nehereus*), Faishya (*Setipinna phasa*), Poa (*Argyrosomus regius*), and Surma (*Scomberomorus guttatus*). Fish drying is mainly done in *Khola* (Bengali name of fish processing facility), where raw fish is spread on bamboo mats on the floor or placed on bamboo scaffolding or shelves for drying.

On the other hand, Chitapara is in Cox’s Bazar town near the Bangladesh Fisheries Development Corporation (BFDC) fishery ghat. In this area, rooftop fish drying practices are carried out on a small scale using bamboo shelves through solar energy. As a result, dried and salted-dried fish and high-value byproducts (fin, swim bladder) are also produced, which are in significant demand from neighboring countries and the tribal communities.

### 2.2. Empirical Data Collection Methods

A total of 250 dried fish processors, workers, traders, and fishers involved in fish drying activities directly or indirectly from Nazirertek (*N* = 215) and Chitapara (*N* = 35) were randomly selected to perform the study from September to December 2019. A mixed approach was applied, including individual interviews (*N* = 250), interviews with key informants (15), and ten focus group discussions (FGD) with checklists (Table 1).

After developing a semi-structured questionnaire, a face-to-face survey was conducted with *N* = 250 respondents to gather qualitative and quantitative information. This study examined several socio-economic indicators hypothesized to reflect the livelihood activities, economic conditions, and food security of people involved in fish drying activities. Through the interviews, a range of qualitative information was obtained by asking communities about livelihood diversity, underlying constraints and vulnerabilities, and mechanisms for coping with the financial crisis and seasonal fluctuations. In addition, direct observations and interviews with key informants, processors, traders, and fish drying workers helped to gather information about the processing and trade of dried fish, the use of preservatives, hygienic and sanitation conditions that constitute a significant concern for public health. Therefore, to analyze these qualitative data, a content analysis method that interprets and encodes different transmitted materials (e.g., documents, articles, books, audios, interviews, and images) through classification, tabulation, and evaluation was employed [21]. Later, all qualitative and quantitative data were entered into Microsoft Excel Spreadsheet 2013 and then analyzed in IBM SPSS version 22, such as descriptive statistics and chi-square test.

### 2.3. Secondary Data Collection

The secondary data were collected from relevant published books, scholarly articles, relevant literature, and newspapers [20] through an online search, e.g., Google scholar.

### 2.4. Sustainable Livelihood Framework

To understand fish drying communities’ resilience based on dependency upon the available assets, this study applied the “Sustainable livelihood approach” (SLA). SLAs are a way of understanding the needs of the poor and identifying the significant constraints and positive strength for their resilience. Based on the sustainable livelihood framework, a fishery-based livelihood embraces several components: (a) livelihood assets (owned or accessed by people, i.e., human, financial, physical, natural, and social capital), (b) vulnerability context (risk factors surrounding livelihoods); (c) transforming structures and processes (the structures associated with a formal organization, e.g., government, NGOs, laws and rights, social relations and participation) (d) livelihood strategies (the range and combination of activities people undertake or do to achieve livelihood goals such as productive activities, investment strategies); and (e) livelihood outcomes (achievement or output of the people’s livelihood strategies) [22]. Through open-ended interviews and FGD, much information was gathered about access to different types of capital, livelihood strategies and decision-making processes, local institutions, and their ability and willingness to respond to changing vulnerability contexts. Themes were identified and classified into manageable categories of different variables: physical capital, financial capital, social capital, strength, threats, and outcomes.

### 2.5. Community Resilience Assessment

In terms of community resilience, the term ‘socio-economic stability’ denotes how the community can maintain their livelihoods and desired living standards without outside support, following undesirable shocks [21]. As the fish drying communities in Cox’s Bazar region are vulnerable to seasonality and extreme weather conditions, it is essential to know how the communities manage resilience in the face of change.

**Figure 2:**
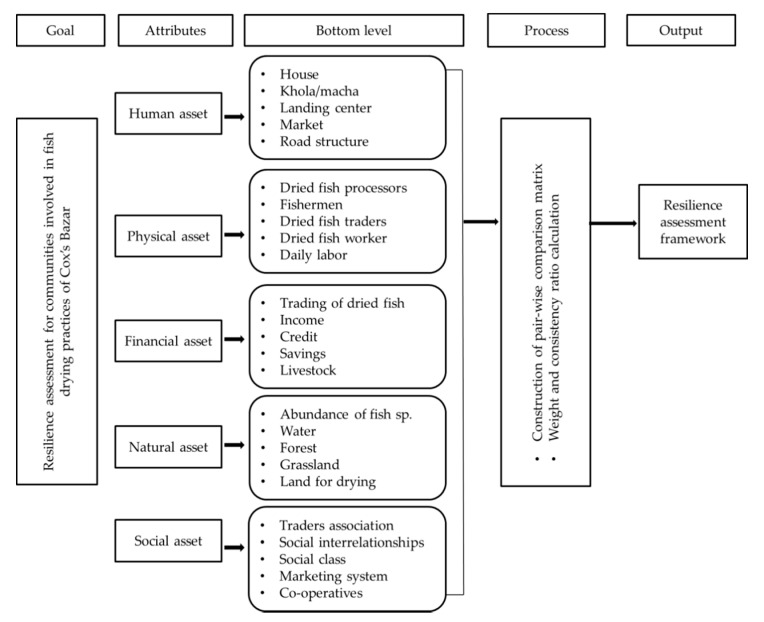
The adapted analytical hierarchy process (AHP) for resilience assessment of communities involved in fish drying practices in the Cox’s Bazar region of Bangladesh.

The procedure for assessing the resilience of fish drying communities is presented in Figure 2. A multi-criteria decision model-the adaptive analytical hierarchy process (AHP) was used for resilience assessment of the communities involved in fish drying (Figure 2). The model structure for assessing communities’ resilience was based on a three-level hierarchical structure that breaks down all criteria into sub-models. To cluster a hierarchy, it was first decided which criteria to group, based on the similarity of these criteria in terms of the functions they perform or the features they share [23].

The top or first hierarchy level represents the goal of the multi-criteria decision-making analysis process. In contrast, the intermediate or second hierarchy level lists the respective evaluation criteria compared pair-wise to assess their relative weight. Each of these clusters was considered a sub-model. Finally, the bottom level of the hierarchy contains criteria for evaluation. All of these criteria (sub-attributes) were identified to create a pair-wise comparison matrix that assesses the relative importance of the different measures to evaluate the resilience of communities involved in fish-drying practices.

#### Weight and Score

The development of weight was based on a pair-wise comparison matrix. A comparison of the relative importance of the two criteria was involved in determining resilience for specified objectives (Table 2). To use this procedure, the weights needed to sum up to 1. Ratings are systematically scored on a 17-point continuous scale from 1/9 (least significant) to 9/9 (most important) [23]. In this research, scores were assigned in rank order according to the number of factors involved in evaluating resilience assessment without repetition. Consistency ratios (CR) of 0.0030 to 0.0145 for the table were well within the balance of less than or equal to the 0.10 recommended by Saaty [23], signifying a small probability that the weights were developed by chance [24].

The present study identified 25 essential criteria of livelihood assets to assess communities’ resilience, such as human assets (dried fish processors, fishermen, dried fish traders, fish drying workers, and fish drying worker), financial assets (trading of dried fish, daily income, credit, savings, and livestock rearing), natural assets (abundance of dried fish species, enough land for drying, water, forest, and grassland), social assets (co-operatives, dried fish traders’ association, social interrelationship, social class, and marketing system), and physical assets (house, *khola/macha*, landing center, market, and road structure) (Figure 2). Weights were given following the effectiveness of the criteria. The weight for each factor was determined by pair-wise comparisons in the context of a decision-making process known as the analytical hierarchy process [23,25], which was also suggested by other authors [21,26,27,28]. The assessment of resilience at each level of the factor was determined from survey findings and professional judgment.

### 2.6. Statistical Analysis

Multinomial logistic regression analysis, Principal Component Analysis (PCA), and community resilience analysis were performed through SPSS Version 22 (IBM) and MS Excel Spreadsheet 2013.

#### 2.6.1. Likelihood Ratio Test

Likelihood ratio tests between various categorical variables (has two or more categories, also known as qualitative variables) indicate a significant association between the variable and the socio-economic state of the community. In this study, descriptive statistics (cross-tabulation analysis) were applied to perform likelihood ratio tests between various categorical variables through SPSS software. The categorical demographic variables such as age, gender, religion, marital status and educational status, occupational profile, family type, training facility, access to resources, food security, food type, and savings scheme were chosen to find significant associations.

#### 2.6.2. Binary Logistic Regression Analysis

A binary logistic regression model was used to examine how socio-economic and demographic variables affect fish drying activities’ food safety. In the study, respondents were considered to have food securities if they were able to intake enough food three times a day. Otherwise, respondents were considered food insecure, if they could not manage enough food for their families in one day or eat twice a day. As the dependent variable (having food security or not) is dichotomous, the model was used to identify the factors affecting the odds ratio of the food status of the communities. The odds ratio refers to people’s probability of having food insecurity (Pi) to predict people’s food insecurity (1 − Pi). The dependent variable used in the study is the dummy variable that takes the value of one for having (food security); 0 otherwise (food insecure).

The logistic model of the relationship between the respondent’s food security status variable and its explanatory variables is specified as Equation (1):

In[Pi/(1 − Pi)] = β_0_ + β_1_X_1i_ + β_2_X_2i_ +……. (1)

where subscript i denotes the i-th observation in the sample, P is the probability of the outcome, β_0_ is the intercept, and β_1_, β_2_… are the coefficients associated with each explanatory variable, X_1_ (age), X_2_ (Gender), X_3_ (occupation)......

#### 2.6.3. Multinomial Logistic Regression Analysis

A multinomial logistic regression model is generally used when there is a categorical dependent variable where the dependent variable is nominal and has more than two categories. It uses one category as a referenced category (any one of them) and compares other categories with a reference category by taking log odds. This study’s dependent variable was the respondents’ socio-economic status (SES), and the independent variables were the socio-demographic variables. The respondents were ranked into poor, middle, and rich classes to identify the key determinant of the livelihood strategy according to their income, land ownership, and utility services. Empirically, the MLR in this study can be expressed as Equation (2):

(2) $$\mathrm{Log}=\frac{\mathrm{Prob}(Y_{i}=j)}{\mathrm{Prob}(Y_{i}=j^{\prime })}=\alpha +\beta_{1}(\mathrm{age})+\beta_{2}(\mathrm{marital})+\beta_{3}(\mathrm{ocupation})+\beta_{4}(\mathrm{house})+\beta_{5}(\mathrm{owner})+\beta_{6}(\mathrm{drinking})+\beta_{7}(\mathrm{income})+\beta_{8}(\mathrm{credit})\ldots \ldots \ldots$$

J is the identified cluster, poor class, and middle class, and j′ is the reference cluster, Rich class.

## 3. Results

### 3.1. Socio-Demographic Profile of the Communities

The main occupations of all respondents in the selected community were fish drying, fishing, and fish trading, and they spent the busiest time in about nine months engaging in drying. During the survey, various age groups of dry fish producers, workers, and traders varying from 5 to 60 years were found to be involved in fish drying where most of them (38% at Nazirertek and 49% at Chitapara) belonged to the age group of 30–40 years. The communities’ religious status revealed that the fish drying business was mainly dominated by Muslims (94%, Nazirertek, and 86%, Chitapara). Simultaneously, the minorities were the Hindu communities who had been seen to be involved in fishing and other businesses (Table 3).

Data obtained from the survey showed that most of Nazirertek (36%) communities had no formal education, whereas in Chitapara, 37% of respondents could only write their names. However, 29% of Nazirertek respondents could only sign, while the other 24% and 11% had primary and secondary education, respectively. In Chitapara, these percentages were 37%, 31% and 3%, respectively. Housing conditions of a community indicate the level of well-being or economic status of the people. In Nazirertek, 42% of respondents lived in houses made of wood with a tin shed, and the other 32% lived in straw roof houses, whereas in Chitapara, 34% lived in buildings, and 51% lived in houses made of tin and wood (Table 3).

Migration is defined as the movement of people from one place to another within the state’s boundary to take up employment or establish residence. The survey showed that 79% of respondents from Nazirertek were migrants, while in Chitapara, the number of migrants was lower (35%) than Nazirertek (Table 3). At Nazirertek, it was found that 44% of respondents did not need any financial help or did not take any loan, whereas 36% of respondents borrowed money from co-operatives, 11% from NGOs or banks, and 9% borrowed from their neighbors. In Chitapara, these percentages were 51%, 15%, 20% and 14%.

### 3.2. Gender Perspectives on the Livelihoods of Fish Drying Communities

**Figure 3:**
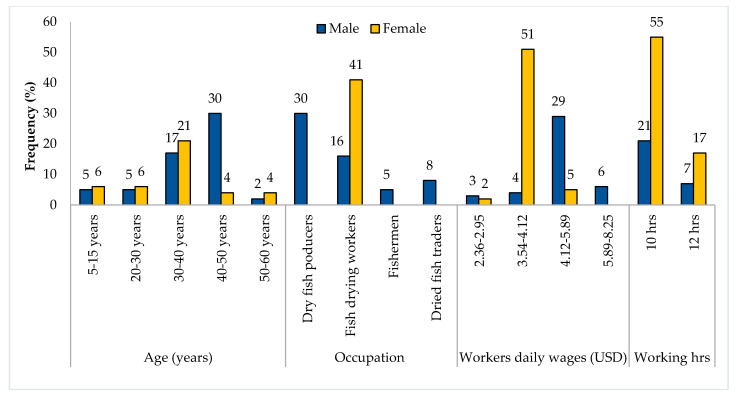
Gender participation profile of the fish drying community living in Nazirertek.

In Nazirertek, most of the fish drying workers (16% of male and 41% of female) were employed by the dry fish producers (30%) and traders (8%), while women (especially widows, divorcee) made up the majority of the dried fish workers. As a result, most male workers (29%) received USD 4.12–5.89 day^−1^, and only a few (6%) received USD 5.89–8.25 day^−1^ by working from 10–12 hrs. On the other hand, more than half of the women (51%) received USD 3.54–4.12 day^−1^, and only 5% received USD 4.12–5.89 day^−1^ (Figure 3).

**Figure 4:**
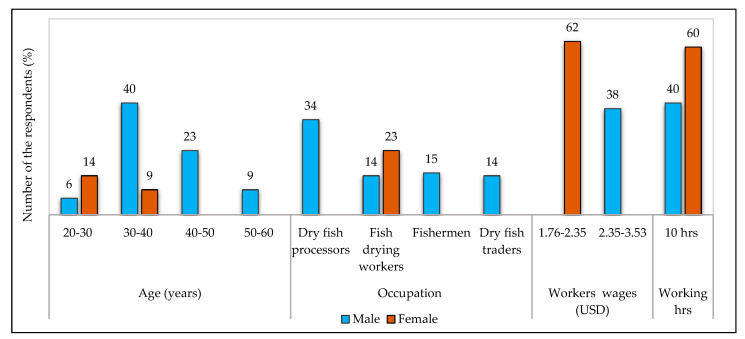
Gender participation profile of the fish drying community living in Chitapara.

Most of the child workers in Nazirertek belonged to the age group 5–15 years (5% male, 6% female), while male children earned USD 2.02–2.52 day^−1^ (3%) to USD 3.54–4.12 day^−1^ (4%) and female earned (2%) USD 2.36–2.95 day^−1^ (Figure 3). Figure 4 shows that no children were engaged in drying fish in Chitapara. In contrast, male workers received maximum wages (62%) ranging from USD 2.35–3.53 day^−1^, and the minimum wages were received by female workers (38%) ranging from USD 1.76–2.35 day^−1^.

### 3.3. Reasons behind Employing Children in Drying

**Figure 5:**
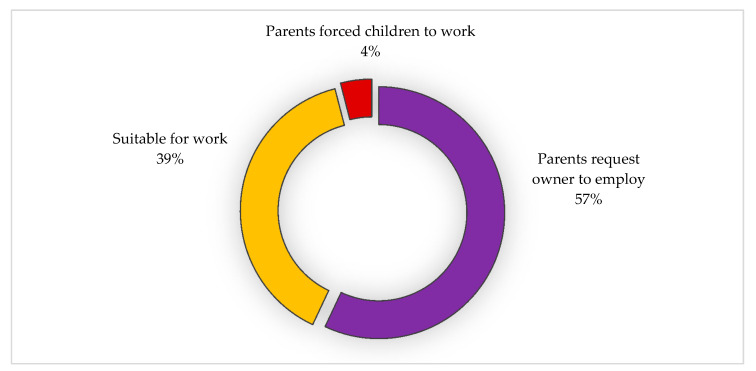
Reasons behind employing children for fish drying activities in the study areas.

Respondents were asked why children were employed, in which 57% of the dried fish processors and owners of Nazirertek reported engaging children at the request of their parents; 39% thought the area was suitable for child labor, and 4% said some parents force their child to work (Figure 5). No children in Chitapara have been found to participate in fish-drying activities, which may be due to their parent’s self-reliance in sustaining their livelihoods.

### 3.4. Health Issues and Treatment Facilities

**Figure 6:**
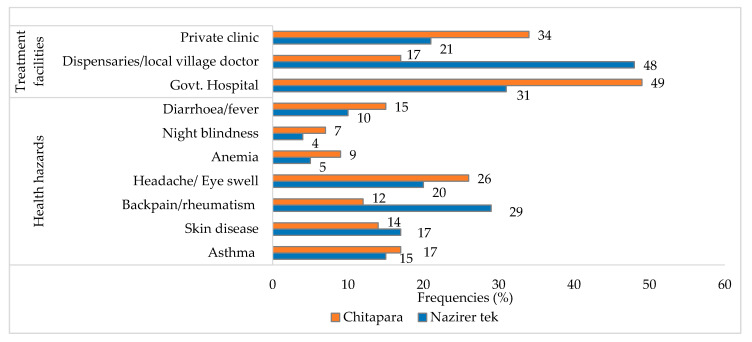
Occupational hazards and treatment facilities of the communities involved in fish drying practices.

About 29% of respondents at Nazirertek reported suffering from back pain/rheumatism, whereas at Chitapara, 26% of respondents reported suffering from swelling of the eyes. In addition, swelling of the eyes, skin disease, asthma, diarrhea or fever, anemia, and night blindness were also reported to be suffered by respondents (Figure 6). Moreover, the health facilities for the communities were deplorable; and 48% of respondents from Nazirertek relied on dispensaries for treatment and 49% of the respondents of Chitapara received treatment from Govt. hospital (Figure 6).

### 3.5. Income during the Drying Season (Peak Season)

**Figure 7:**
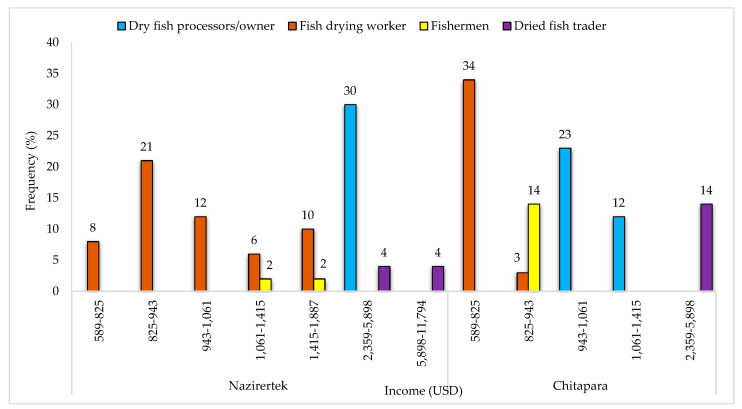
The annual income of the communities according to their occupations.

In the fish drying season, 30% of dried fish producers in Nazirertek earned USD 2359–5898 year^−1^, whereas the highest income was earned by dried fish traders (4%), varying from USD 5898–11,794 year^−1^. Among the fish drying workers, 21% earned USD 825–943 year^−1^, whereas fishermen’s annual income ranged from USD 1061–1187 year^−1^ (Figure 7). In Chitapara, fish drying communities run the business on a small scale, so their income was not very high compared to Naziertek, dried fish traders, or producers.

### 3.6. Food Consumption and Security Status

The intake of a poor-quality diet was relatively higher in Nazirertek than in Chitapara (Table 4). The study revealed that about 95% and 86% of the respondents, both from Nazirertek and Chitapara, were able to take three meals daily, whereas the poor workers had to skip a meal a day during the off-season. The situation of food security between the two villages was relatively higher in Chitapara than in Nazirertek.

Binary logistic regression analysis gave the most suitable model with seven variables (age, gender, alternative occupation, household size, income, sole earning member of the family, and house structure) that significantly impact communities’ food security. As shown in Table 5, the age of the respondents has a positive coefficient (0.744) and a significant effect (*p* < 0.05 level) in ensuring the food security status of the communities. This indicates that very old respondents are more likely to have food security, which may be due to their work experience accumulated with age. In addition, the results showed that having an alternative occupation was positive and significant at the (*p* < 0.05) level. This specifies that the higher the alternative income-earning activities, the higher is the probability that the community would be food secure. Hence, a unit increase in alternative income levels will increase communities’ likelihood of being food secure by 9.528.

The likelihood that a household will have food security depends on the source of household income. The result showed that the annual income of communities has a significant (found significant at the *p* < 0.05 level) effect on safeguarding food security in the communities with a positive coefficient (0.879). A unit increase in income level increases respondents’ probability of becoming food secure up to 2.408. Having a single earning family member has a negative significant association (significant at the *p* < 0.05 level) to ensuring food security for the dried fish communities. The results showed that a decrease in the family’s earning members would increase food insecurity. The housing structure had a negative coefficient (−0.614), which had little effect on food security, but it was found to be significant at the (*p* < 0.05) level. Table 5 showed that people living in tin, wood, thatched-roof, and bamboo houses are less likely to have food security than those living in buildings and semi-pacca houses. This may be that most people, especially workers living in bamboo and straw-roofed homes, must pay rent during the drying season due to most of their income being spent buying food items and on rental houses.

### 3.7. Public Health Concern

**Figure 8:**
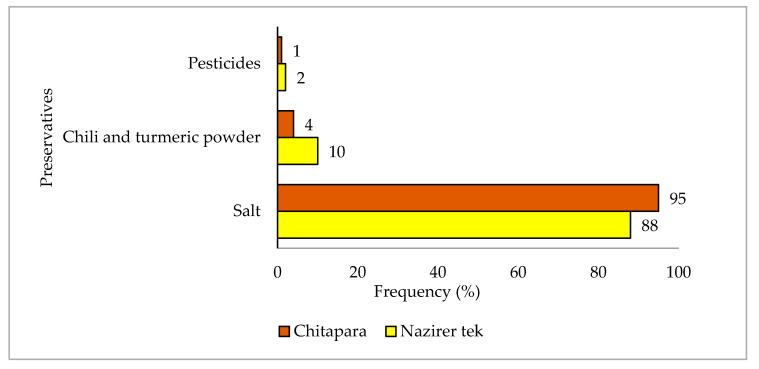
Frequency of using preservatives during fish drying and processing.

During the study period, the rate of using pesticides to dry fish was relatively low (2% at Nazirertek and 1% at Chitapara); on the other hand, pre-processing of raw fish during drying was unhygienic (Figure 8). For drying, low-value fish are first brought from the fish landing center to the drying facility (*Khola*). The fish are then washed with water as needed and dumped on bamboo mats. Fish are then mixed with salt before being sorted, which is when the fish’s quality significantly deteriorates. After sorting, the fish are washed with water in a bamboo basket or plastic bucket or drums, and spread on a bamboo mat or shelf to dry. Unfortunately, the bamboo mats and baskets used are often dirty and are not washed after one drying cycle and before drying the next batch. Apart from public health concerns, such conditions promote the attack of blowflies that infest fish during drying, especially in the rainy season when rain makes drying difficult.

### 3.8. Livelihood Constraints and Vulnerability Context

**Figure 9:**
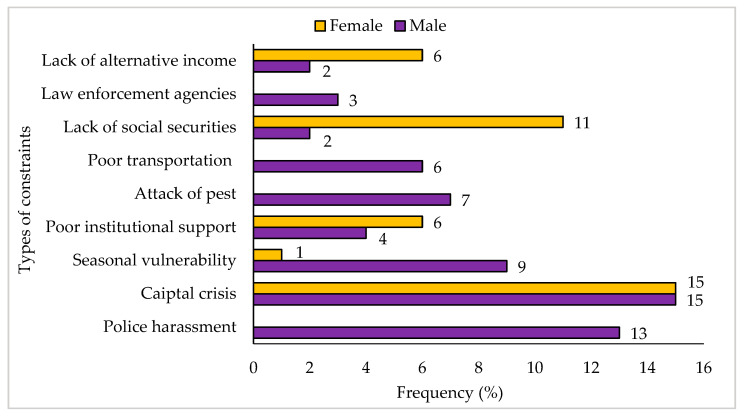
Significant constraints to diversified livelihoods faced by male and female respondents of the community.

As the fish drying communities on the south-east coast are highly vulnerable to seasonality and extreme weather conditions, these people face many socio-economic constraints to sustaining their livelihoods, such as capital crisis, lack of social securities, and poor institutional support for borrowing money (Figure 9).

### 3.9. Coping Mechanisms in the Off-Season

**Figure 10:**
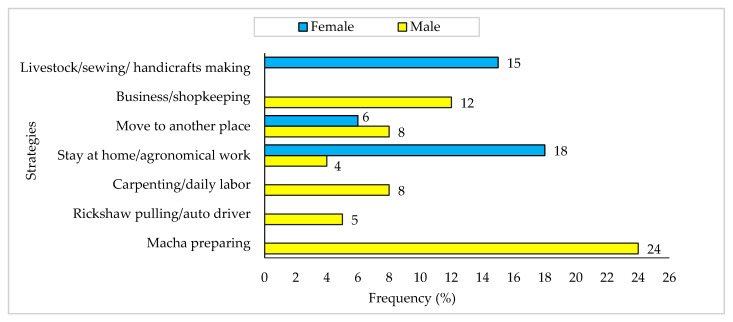
Coping mechanisms evolved by the communities during the off-season.

Fish drying practices were mainly carried out from August to April (called peak season), and during these nine months, the traders, processors, and workers were found to pass time by drying fish. However, during the monsoon season (also known as the off-season), there was no fish drying activity. In the off-season, the situation became unbearable where both men and women pursued different initiatives to support their livelihoods. Figure 10 illustrates that about 4% of men and 18% of women stay at home and do agronomical work. In comparison, 24% of the male respondents prepared *Macha* (Bengali name of fish drying facility) for drying fish, and 15% of the female respondents spent time dedicating themselves to raising poultry, sewing clothes, and doing handicrafts.

### 3.10. Assessment of Resilience to Communities’ Livelihoods Vulnerabilities

**Figure 11:**
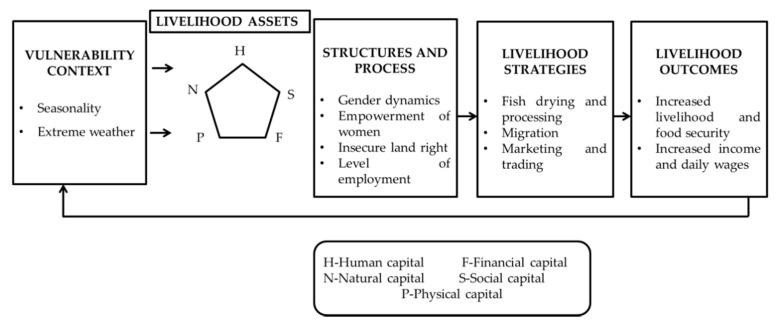
Sustainable livelihood framework of the communities.

This study applied the ‘Sustainable Livelihood Framework’ to understand the resilience of communities based on the level of dependence on existing assets (Figure 11). In assessing resilience, the identified human assets were the dried fish processors, fishermen, dried fish traders, fish drying workers, and daily labor, managing the fish drying communities with their experiences and professional knowledge. The indicators of physical assets were houses, *Macha* for fish drying, fish landing center, markets, and road structures, and the loss of these physical assets leads to complete suspension of fish drying activities.

The availability of dried fish species and enough land for drying were the communities’ main livelihood options. The other natural resources that played a vital role in the communities’ resilience were water, forest, and grassland, whereas grassland protects against land erosion. Production and trading of dried fish, daily income, and food expenditure were identified as critical financial assets that play an indispensable role in community resilience. However, the role of credit, illegal tax, and livestock cannot be ignored. The communities’ social assets, including co-operatives, dried fish traders’ association, social interrelationship, and social class, play an essential role in maintaining economic growth and human well-being (Table 6).

**Figure 12:**
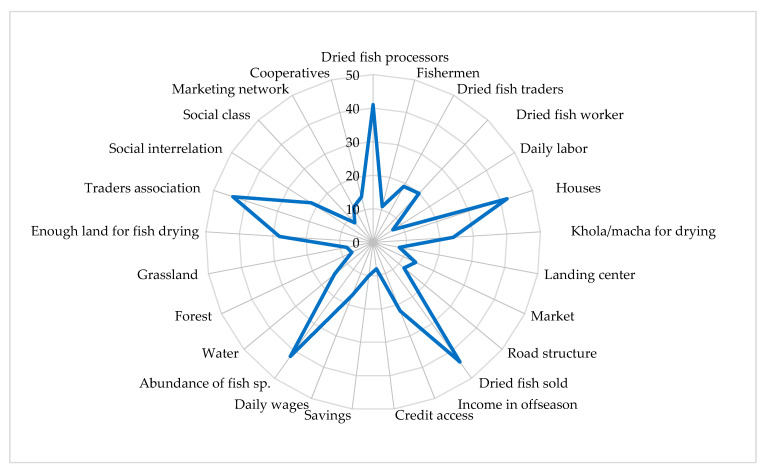
The relative importance (%) of livelihood assets in determining the community’s resilience.

The effectiveness of sub-attributes in each asset is summarized in Figure 12. The results showed that the criteria of dried fish processors, dried fish workers, house, *khola/macha* for drying, selling of dried fish, income during the off-season, and the abundance of fish species, enough land for fish drying, trader’s association, and social interrelationship were relatively high. Additionally, those criteria are effective by 20–40% compared to other livelihood assets that indicate the most increased role in resilience assessment, whereas fishers, dried fish traders, market, road structure, savings, livestock, water, marketing network, and co-operatives were found with 10–19% relative effectiveness, indicating a moderate role in building resiliency. The sub-attributes of daily labor, landing center, credit access, forest, grassland, and social class had less than 10% relative effectiveness, indicating the least significant resilience assessment.

### 3.11. Likelihood Ratio Test between Different Pairs of the Categorical Variable

Likelihood-ratio tests between categorical variables showed that all other variables, except religion and alternative occupation, had a significant association (1%, 5%) with the community’s socio-economic status (Table 7).

### 3.12. Multinomial Logistic Regression Analysis According to Socio-Economic Status

This analysis was accustomed to determining whether demographic variables influence poor, middle, and rich class communities’ livelihood strategies. Table 8 represents the parameter estimate for the final mode while the “Rich class” category had been taken in the reference group. The odds ratio coefficients of the model’s demographic variables were calculated, and then the estimated coefficients of the two classes were compared with the reference category. The results from the logistic analysis indicated that out of 15 hypothesized variables, three socio-demographic variables (drinking water facilities, income in the peak season, and credit access) were found to have a significant influence on the livelihood strategies of the “Poor class” respondents and seven variables (education, sole earning member of the family, having children, drinking water and treatment facilities, income in the peak season and credit access) had a significant influence on the livelihood strategies of the “Middle class” communities at 1%, 5%, significance level. When compared with the other demographic variables, the income of the poor class respondents had an Odds Ratio (OR) = 0.007 (95% CI 0.000 to 0.300), *p* = 0.010 and credit access to services had OR = 20.389 (95% CI 2.613 to 159.060), *p* = 0.004; drinking water facilities had an Odds Ratio (OR) = 0.003 (95% CI 0.002 to 0.267), *p* = 0.011. From the “middle class” categories, the results showed that the educational status of the respondents had an Odds Ratio (OR) = 2.424 (95% CI 0.982 to 5.985), *p* = 0.055; sole earning member of the family had Odds Ratio (OR) = 1.240 (95% CI 0.943 to 1.631), *p* = 0.124; number of children had an Odds Ratio (OR) = 8.155 (95% CI 1.426 to 46.651); income of the respondents had Odds Ratio (OR) = 0.399 (95% CI 0.171 to 0.930), *p* = 0.033; drinking water facilities had an Odds Ratio (OR) = 110 (95% CI 0.020 to 0.592), *p* = 0.010; treatment facilities of the respondents had an Odds Ratio (OR) = 0.155 (95% CI 0.063 to 0378), *p* = 0.142; and credit access to services had an OR = 4.277 (95% CI 1.658 to 11.034), *p* = 0.003.

## 4. Discussion

The availability of marine fish, and the drying, processing, and trade of these fish have brought solvency to many poor coastal populations and increased socio-economic well-being. However, they also exposed them to many constraints that drive the need to improve community resilience. The communities engaged in fish drying activities on the south-east coast are socio-economically backward due to their labor-intensive occupation, lower literacy level, unstable income, dependency on seasonal drying, lack of access to resources, and alternative income. Due to the relatively low education rate in the dry fish community, people have always been lagging in improving sustainable livelihoods, income diversification, modern technology adaptation, and socio-economic welfare. Among the two villages, the illiteracy rate was relatively higher (36%) in Nazirertek than in Chitapara (29%). At the same time, more than one-third of the respondents in Chitapara could only write their names (Table 3). This result coincides with the study conducted by [29], which expressed that 25% of dried fish producers in Barisal and 40% of dried fish producers in the Kuakata region were uneducated. While people once had the myth that more families could earn more money [30] due to poverty and daily expenses, most community members in the present study prefer to have a nuclear family rather than a joint family. Household profiles of fish-drying communities on the southeastern coast revealed that most people had to manage a large family of 5–7 people dominated by adult members, exceeding the national average household size (4.5 people per household). This result is consistent with the study of [31], reporting that half of the fishermen in the Noakhali area belong to 5–6 families.

The participation of women among workers was higher than men (Figure 4 and Figure 5). Many widows and divorcee women had become self-reliant by working in the fish drying yards. Fish drying workers were employed seasonally by producers and traders, while some worked as permanent workers and casual workers. Men managed virtually all the dried fish processing activities, but most of the work was done by female workers, such as tying pairs of dried fish and grading, sorting, and rotating dried fish. More or less similar results were reported by Almaden [16], where the author noted that women were mainly engaged in fish processing and men were involved in catching fish. During the study, females were asked if they were more discriminated against than men. In that case, a mixed reaction was observed among them, and some replied they were subjected to discrimination, while others said that men were doing heavier work than women and get paid more than women. A study conducted by Roy et al. [18] reported that gender discrimination was widespread among Indian Sundarban.

The housing pattern, ownership of land, and houses is an important indicator to assess the socio-economic well-being of a community. It was observed that half of the respondents (50%) of Nazirertek dwell in rented houses, where 72% of traders and processors in Chitapara had their own houses to live in (Table 3). The causal workers of Nazirertek stated that during the peak season, they lived in poorly constructed rental houses near the drying sites built or leased out by the owners/traders. They also revealed that they found it challenging to share a single room with their 5–6 family members and pay USD 7.07–8.26/month, whereas this scenario was less in Chitapara. On the contrary, it specified that 78% of fry and fingerling traders had their own houses, and 22% lived in rental houses [32].

Access to sanitary, clean, and safe drinking water is regarded as an essential fundamental in society. Despite the local authorities trying to secure basic facilities, the community’s basic facilities were less than satisfactory. Most of the study areas’ communities have electricity facilities, but the other facilities (drinking, sanitary) enjoyed by the Nazirertek communities were not as good as Chitapara. Asif et al. [32] pointed out that the sanitation facility of traders in the Jessore region was good. However, not all fishermen on Nijhum Dip Island have access to electricity facilities and instead use solar power [33]. Therefore, these results do not correlate with the current findings.

Migration is an essential determinant of livelihood strategies. The study revealed that most producers or traders were displaced by natural disasters from Kutubdia, Chakaria, Moheshkhali, Myanmar (Rohingyas) (Table 3) and lived in Nazirertek permanently for 15 to 32 years. During peak season, seasonal fish-drying workers also move to Nazirertek, searching for work with their families and living in houses built by processors and traders near the drying area. Therefore, seasonal fluctuations are not the only factor driving communities to migrate. Further, different migration factors such as lack of employment opportunities and social insecurity in the local community force them to move elsewhere. Similar observation stated that fishers and their families on the south-central coast pushed highly to nearby places due to socio-economic vulnerabilities, insecurities, and unemployment [30].

Most of the communities had been observed to work long hours in the hot sun. As a result, they suffered from various health issues, including headache/swelling of the eyes, back pain, rheumatism, and dark skin due to the seasonal changes and exposure to sunlight (Figure 6). There was one Upazila Health Complex in the study area, but the medical facilities the community enjoyed were unsatisfactory as they had little capacity to pay for medical care. According to the study, more than one-third of respondents, as dry fish producers, traders, and fishers, had access to a fishery office (Table 3), institutional organization, sea, market, and firewood. In contrast, most of the fish drying workers had no access to an institutional organization or microcredit access due to their social status in the communities. Singh et al. [34] reported that about 82.50% of Coastal Odisha women had access to the market, and less than one-third of males and females had access to institutional credit. Thus, these findings are not relevant to the present study.

Among the people involved in drying, dried fish producers and traders had better livelihoods than workers due to their high annual incomes, occupation, ownership of their assets, and seasonal investments in fish drying. However, most of the workers were landless and poor and were exploited by producers and traders while working under the supervision of producers and traders. Belton et al. [1] also made a similar observation, who noted that workers from very different social origins were employed in fish drying under various production relations mixtures. The authors also concluded that this had a significant impact on workers’ lives but often led to the exploitation of subgroup workers, which adversely affected social welfare.

Communities engaged in fish drying activities were found to work hard throughout the day to manage their food and livelihoods. However, most of them had difficulty meeting the necessities of life. As the income of dry fish producers or traders was high (Figure 7), it was easier for them to meet their livelihoods and basic requirements. On the contrary, much of the income of poor fishermen and workers was spent managing food, treatment, and education. They were found to suffer from food shortages, and to withstand this situation they were compelled to reduce their meal frequency to two meals per day and try to consume less-expensive food items (Table 4). Reducing meal frequency and fish consumption reflects their low-income level and lack of alternative livelihood opportunities during the off-season. Similar findings were also reported by Rana et al., Mondal et al. [17,35].

The age of the respondents, having an alternative occupation, income, sole earning member of the family, and house structure affects the community’s food security in many ways (Table 5). Income is an essential component of economic access to food at both the communities and individual levels. From Table 5, it can be concluded that the higher the annual income of the participants, the more likely the participant has food security. In the off-season, most people were out of work because drying and processing were their main occupations; nevertheless, they had to rely on other alternative professions to maintain their livelihoods and manage their food. Hence, earning family members also affect the food security status of the communities. Simultaneously, the more earners they have in the family, the more money they can achieve and the more they can manage their families. Thus, the community will have more food security. A similar observation was made by Omotayo and Aremu [36], showing that age, gender, and household size significantly impact food security status among rural households in the North West State of South Africa. Moreover, this study’s findings also correspond to Maharjan and Joshi [37], who concluded that providing income-generating opportunities to economically active age people can significantly reduce food insecurity.

Fish drying activities in the study areas were highly seasonal, and most of the respondents, especially the fish drying workers, were employed on a seasonal basis. This pattern and level of employment harmed community livelihoods, including capital crisis and social insecurities in the workplace (Figure 10). According to Marimuthu [38], the inland fishermen were found to face many problems such as employment patterns, lack of transportation facility, no safety, or high risk, which was more or less similar to the present study. Furthermore, most of the communities largely depended on fish drying activities. However, during the off-season, the communities’ situation became so unbearable that both men and women had to pursue different initiatives to support their livelihoods (Figure 10). To cope with the financial crisis, they had to borrow money from various institutions and relatives for livelihood maintenance or other purposes, including marriages, festivals, medical, and different basic needs [39,40].

Community resilience embraces the idea of how biophysical and socio-economic systems can respond to any changes, unpleasant shocks, and seasonality [28]. As solar energy is the fundamental element to drying fish, harsh weather environments such as windstorms, heavy rain, cloudy days, and destructive current can be a significant barrier to the communities’ resilience. The resilience study revealed that the fish drying communities were less resilient to livelihood vulnerabilities than other communities because they relied on natural resources, solar energy, and migration (Figure 12). The dry fish processors play a crucial role in maintaining resilience, where the workers work as ‘food for work, cash for work.’ In the case of community resilience, the experienced and skilled dry fish producers and workers, production and trading of dried fish, enough land for fish drying, availability of dried fish species, traders’ association, and social interrelationship among the communities has been found to play a crucial role in enhancing community resilience (Table 6). Hossain et al. [24] also made a similar observation on the resilience assessment of fishing communities at Nijhum Dwip Island.

As far as quality control and public health are concerned, it has been observed that most of the processors, traders, and workers throughout the study period use salt, chili, and turmeric powder as preservatives rather than pesticides to dry and process fish. Although the extent of pesticide application has been reduced, only a tiny part of the community has reported spraying pesticides (*Sobicron, Nogos*) to the dried fish to reduce pest attacks on heavy rains and cloudy days. The residual effects of these pesticides can be very detrimental to human health. So far, dried fish cannot reach a broader range of wealthy consumers, especially health-conscious consumers.

When conducting the study, the main limitations were dialect issues and bad weather conditions, which made communication with the respondents very difficult. Therefore, the most fundamental challenge for the dried fish sector will be ensuring more sustainable fisheries management. Other challenges in this sector include the insecurity of ownership faced by many drying processors or traders due to their vulnerability to climate change. Fish drying activities in Nazirertek are carried out on *khas* (government-owned land), which can be evacuated due to economic activities and may affect communities’ working and living conditions largely dependent on drying. On the other hand, dried fish for preparing fish meals in aquaculture feed can play a significant role in the fish feed industry.

A more direct approach should focus on diversification of livelihoods and access to basic amenities, including health care, education, clean drinking water, hygiene improvement, and dietary supplements to sustain the livelihoods of communities. Moreover, the local community leader should organize alternative employment opportunities or training facilities for workers, especially in the off-season, to diversify their livelihoods. A safe place should be provided for the dried fish producers and traders to expand their fish drying activities, such as a private zone. Governments with local authorities need to take the necessary steps to increase dried fish exports in domestic and international markets. In addition, processors and workers who follow traditional fish drying techniques should be trained for improved sun drying, hygiene, and public health.

## 5. Conclusions

Across the country, the poor and marginalized coastal people are involved in drying fish and work hard to meet the growing demands of dried fish with their skill and flawless efforts. Nevertheless, their livelihood patterns are less diversified and rarely recognized. Most widows, divorcees, and unmarried female workers were found to be self-reliant on the southeastern coast by working in fish drying yards. Moreover, most workers involved in dry fishing experienced livelihood insecurity as they are landless, poor, and unskilled with a limited work environment and exploited by processors and traders. To boost the community resilience of the dry fish workers, it is vital to expand alternative occupation and social protection, providing community empowerment for making resource-use decisions and institutional, organizational, and government support. Therefore, this study’s findings will contribute as a base knowledge for government and local management authorities for new, practical, and equitable management for communities.

## Figures and Tables

**Table 1:** An overview of empirical data collection methods.

Tools	Participants	Sample Size	Research Issues/Objectives
Individual interview (II)	Dried fish traders, processors, workers, fishers involved in fish drying practices	*N* = 250 Male-153 Female-97	Socio-demographic factors- age, religion, gender, marital status, family type, household size, education, occupation, housing condition, income, savings, credit access, etc.
Key informant interview (KII)	Members of dried fish trader’s association, knowledgeable persons in the communities, Fisheries Scientific Officers, NGO workers.	15	Knowledge and experience persons often play a vital role in the community. Cross-checked interviews validate the collected data.
Focus group discussion (FGD)	Elderly and young male and female workers, community leaders, widow, experienced fishermen	10 (5–8 participants)	Semi-structured data gathering method that takes advantage of group dynamics and allows respondents to discuss critical issues
PRA tools	Male and female respondents of the community	20	Daily activity chart, seasonal variations

**Table 2:** The relative importance of two criteria [23].

1/9	1/8	1/7	1/6	1/5	1/4	1/3	1/2	1		2	3	4	5	6	7	8	9
Extremely		Very strongly		Strongly		Moderately		Equally		Moderately		Strongly		Very strongly		Extremely	
Less important									More important								

**Table 3:** Summary of the communities’ socio-demographic profile involved in fish drying activities in the study areas (*N* = 250).

Characteristics	Categories	Nazirertek (*n* = 215)	Chitapara (*n* = 35)
		Frequency (%)	Frequency (%)
Household profile			
Age (years)	5–20	23 (11)	-
	20–30	24 (11)	7 (20)
	30–40	82 (38)	17 (49)
	40–50	73 (34)	8 (22)
	50–60	13 (6)	3 (9)
Religion status	Hindu	13 (6)	5 (14)
	Muslim	202 (94)	30 (86)
Marital status	Married	123 (57)	28 (80)
	Unmarried	36 (17)	2 (6)
	Divorced	11 (5)	2 (6)
	Widowed	45 (21)	3 (8)
Occupational profile	Dry fish processors/owner	64 (30)	12 (35)
	Fish drying worker	123 (57)	13 (37)
	Fishermen	10 (5)	5 (14)
	Dried fish traders	18 (8)	5 (14)
Level of education	Illiterate	78 (36)	10 (29)
	Can sign only	62 (29)	13 (37)
	Primary	51 (24)	11 (31)
	Secondary	24 (11)	1 (3)
Family type	Joint	88 (41)	13 (37)
	Nuclear	127 (59)	22 (63)
Family size	A small family (2 to 4)	62 (29)	10 (29)
	Medium family (5 to 7)	125 (58)	14 (40)
	Large family (8 to 10)	22 (10)	7 (20)
	Very large family (above 10)	6 (3)	4 (11)
Number of children	1 to 2	101 (47)	10 (29)
	3 to 4	108 (50)	23 (65)
	5 to 6	6 (3)	2 (6)
Children going to school/not	School going children	123 (57)	31 (89)
	Non-going children	92 (43)	4 (11)
Earning member of the family	One	92 (43)	10 (29)
	Two	58 (27)	23 (65)
	Three	65 (30)	2 (6)
Residential status	Migrant	171 (79)	12 (35)
	Non-migrant	44 (21)	23 (65)
Having an alternative occupation	Yes	24 (11)	9 (26)
	No	191 (89)	26 (74)
Housing and basic facilities			
Housing structure	Buildings	11 (5)	12 (34)
	Semi pacca	45 (21)	3 (9)
	Tin & wood	90 (42)	18 (51)
	Straw roof & bamboo	69 (32)	2 (6)
Sanitary facilities	Pacca	11 (5)	4 (12)
	Open/kacha	19 (9)	4 (11)
	Pit latrine	105 (49)	8 (23)
	Semi pacca/pacca	80 (37)	19 (54)
Drinking water facility	Govt. tube well	136 (63)	14 (40)
	Own tube well	79 (37)	21 (60)
Electricity facilities	Yes	194 (90)	32 (91)
	No	21 (10)	3 (9)
Having social securities (Insurance)	Yes	17 (8)	6 (17)
	No	198 (92)	29 (83)
Ownership of house and land			
Ownership of the house	Owner	90 (42)	25 (72)
	Rented	108 (50)	7 (20)
	Leased	17 (8)	3 (8)
Agricultural land ownership	Less than 5 decimal	37 (17)	4 (11)
	No land	178 (83)	31 (89)
Access to common property resources			
Have access to other resources	Yes	77 (36)	16 (46)
	No	138 (64)	19 (54)
Credit access	Self-sufficient	95 (44)	18 (51)
	Borrowed from NGOs/Bank	24 (11)	5 (15)
	Borrowed from co-operatives	77 (36)	7 (20)
	Borrowed from Neighbors	19 (9)	5 (14)
Participation in training programs	Yes	65 (30)	9 (26)
	No	150 (70)	26 (74)

**Table 4:** Nutrition and food consumption ratio of the respondents of the studied areas.

Types	Variables	Nazirertek (*n* = 215)	Chitapara (*n* = 35)	Remarks
		Frequency (%)	Frequency (%)	
Meal (times/day)	Three times/day	205 (95)	30 (86)	During the off-season, most of the workers had to skip one meal in a day and took two meals/day During the off-season, most of the workers had to take low quality fish and vegetables six days/week and rarely ate meat/milk/egg
	Four times/day	10 (5)	5 (14)	
Having Food security	No	114 (53)	9 (26)	
	Yes	101 (47)	26 (74)	
Variation of food intake daily	Nutritious diet (Rice, fish/meat, vegetables/pulses/egg/milk)	85 (40)	19 (54)	
	Poor quality diet (Rice, low-quality fish, pulses, vegetables, meat once/twice a month)	130 (60)	16 (29)	

**Table 5:** Determinants of the factors affecting the food security of people participating in fish drying activities.

Socio-Demographic Factors		Co-Efficient B	S.E.	Wald	Sig.	Exp (B)
Step 1	Age of the respondents	0.744	0.321	5.365	0.021	2.103
	Gender participation profile	−1.705	0.921	3.426	0.064	0.182
	Occupation	−0.648	0.730	0.787	0.375	0.523
	Alternative occupation	2.254	0.953	5.592	0.018	9.528
	Education	−0.361	0.451	0.639	0.424	0.697
	Household size	0.793	0.509	2.422	0.120	2.210
	Ownership of the house	−0.603	0.735	0.674	0.412	0.547
	Having Livestock	0.458	0.631	0.527	0.468	1.581
	Income during peak season	0.879	0.363	5.856	0.016	2.408
	Credit access	0.048	0.261	0.033	0.855	1.049
	Sole earning member of the family	−1.724	0.799	4.652	0.031	0.178
	Social status	1.007	1.076	0.876	0.349	2.737
	Marital status	0.126	0.309	0.166	0.684	1.134
	Housing structure	−0.614	0.293	4.407	0.036	0.541
	Constant	−3.568	4.108	0.754	0.385	0.028
Significant at, 5%, 1%.						

**Table 6:** A pair-wise comparison matrix for assessing the relative importance of different criteria for resilience assessment of communities involved in fish drying practices to livelihood vulnerabilities in the study areas (numbers show the row factor rating close to the column factor).

Assets/Capitals	Stakeholders Involved in Fish Drying Practices					Weight
Human capital	Dried fish processors	Fishermen	Dried fish traders	Dried fish laborer	Daily labor	
Dried fish processors	1	1/4	1/3	1/2	1/5	0.065
Fishermen	4	1	2	1/2	1/3	0.201
Dried fish traders	3	1/2	1	1/3	1/2	0.190
Dried fish worker	2	1/3	3	1	1/5	0.126
Daily labor	5	2	2	3	1	0.417
Consistency ratio (C.R): 0.0083						
Physical capital	House	Khola/macha for drying	Landing center	Market structure	Road structure	
House	1	1/2	1/4	1/3	1/5	0.066
Khola/macha for drying	2	1	2/3	1/4	1/2	0.132
Landing center	4	2	1	3	2	0.386
Market structure	3	2	1/6	1	1/2	0.162
Road structure	5	3	2/3	1/2	1	0.253
Consistency ratio (C.R): 0.0145						
Financial capital	Dried fish sold	Income during offseason	Credits	Illegal tax	Livestock	
Selling of dried fish	1	1/2	1/3	1/4	1/5	0.073
Income during offseason	2	1	1/2	1/3	1/2	0.128
Credit access	3	2	1	3/2	2	0.327
Illegal tax	4	2	1/3	1	2	0.260
Livestock	5	2	1/2	1/3	1	0.212
Consistency ratio (C.R): 0.0055						
Natural capital	Fish abundance	Water	Forest	Grassland	Enough land for fish drying	
The abundance of fish species	1	1/2	1/4	1/6	1/3	0.067
Water	2	1	2/3	2	3	0.202
Forest	4	2	1	1	2	0.301
Grassland	6	2	1/2	1	4	0.327
Enough land for fish drying	3	1/4	1/5	1/3	1	0.103
Consistency ratio (C.R): 0.0140						
Social capital	Traders association	Social interrelation	Social class	Marketing system	Co-operatives	
Traders association	1	1/2	1/4	1/3	1/5	0.069
Social interrelationship	2	1	2/3	1/2	1/2	0.146
Social class	4	2	1	3	2	0.358
Marketing system	3	2	1/4	1	1	0.202
Co-operatives	5	2	2/3	1/3	1	0.226
Consistency ratio (C.R) 0.0089						
Overall	Human	Physical	Financial	Natural	Social	
Human	1	1/2	1	2	1/3	0.136
Physical	2	1	3	5	1/2	0.301
Financial	1	1/3	1	2	1/2	0.140
Natural	1/2	1/4	1/3	1	1/4	0.070
Social	3	1 1/2	2	4	1	0.353
Consistency ratio (C.R) 0.0030						

**Table 7:** Likelihood ratio test between different categorical variables.

Categories	Value	df	Significant
Age of the respondents	120.738	8	0.000
Gender	156.561	2	0.000
Religion status	0.509	2	0.775
Marital status	128.494	6	0.000
Occupational profile	326.315	6	0.000
Alternative occupation	4.796	2	0.051
Family type	13.901	2	0.001
Residential status	13.650	2	0.001
Level of education	81.302	6	0.000
Ownership of houses	239.006	4	0.000
Electricity facilities	12.828	2	0.002
Drinking water facilities	213.039	2	0.000
Agricultural land	6.103	2	0.047
Having Livestock	11.943	2	0.003
Subsistence Production	11.337	2	0.003
Annual Income (USD)	333.197	12	0.000
Training facilities	14.700	2	0.001

**Table 8:** Multinomial logistic regression analysis according to the economic status of the studied areas.

Parameter Estimates							
Socio-Economic Status		B Coefficient	Std. Error	Sig.	Exp (B) Odd Ratio	95% Confidence Interval for Exp (B)	
						Lower Bound	Upper Bound
Poor class	Intercept	−4.281	11.076	0.699			
	Age of the respondents	2.232	1.766	0.206	9.315	0.292	296.953
	Marital status	3.491	2.452	0.154	32.811	0.269	4006.702
	Occupation of the respondents	0.661	2.073	0.750	1.938	0.033	112.612
	Level of education	−0.044	0.941	0.962	0.956	0.151	6.045
	Sole earning member of the family	−0.707	0.612	0.248	0.493	0.149	1.635
	Housing structure	−0.094	1.125	0.933	0.910	0.100	8.257
	Ownership of the house	−2.147	2.894	0.458	0.117	0.000	33.973
	Number of children	3.148	2.227	0.157	23.297	0.296	1832.378
	School going children	3.366	1.809	0.063	28.964	0.835	1004.358
	Drinking water facilities	−5.728	2.250	0.011	0.003	0.002	0.267
	Sanitary facilities	1.793	1.220	0.142	6.007	0.549	65.690
	Types of disease	0.609	.982	0.535	1.838	0.268	12.609
	Treatment facilities	0.897	2.012	0.656	2.453	0.048	126.644
	Income in peak season	−4.948	1.910	0.010	0.007	0.000	0.300
	Credit access	3.015	1.048	0.004	20.389	2.613	159.060
Middle class	Intercept	−0.894	4.715	0.850			
	Age of the respondents	1.097	0.759	0.148	2.995	0.677	13.245
	Marital status	2.555	2.330	0.273	12.870	0.134	1237.758
	Occupation of the respondents	0.103	0.329	0.753	1.109	0.582	2.112
	Level of education	0.885	0.461	0.055	2.424	0.982	5.985
	Sole earning member of the family	0.320	0.157	0.042	1.377	1.011	1.874
	Housing structure	0.066	0.252	0.792	1.069	0.653	1.750
	Ownership of the house	−2.469	1.677	0.141	0.085	0.003	2.267
	Number of children	2.099	0.890	0.018	8.155	1.426	46.651
	School going children	0.963	0.641	0.133	2.620	0.746	9.202
	Drinking water facilities	−2.207	0.859	0.010	0.110	0.020	0.592
	Sanitary facilities	0.431	0.410	0.293	1.539	0.689	3.437
	Types of disease	−0.164	0.296	0.579	0.849	0.476	1.515
	Treatment facilities	−1.866	0.456	0.000	0.155	0.063	0.378
	Income in peak season	−0.919	0.432	0.033	0.399	0.171	0.930
	Credit access	1.453	0.484	0.003	4.277	1.658	11.034
